# Immunophenotype of Kawasaki Disease: Insights into Pathogenesis and Treatment Response

**DOI:** 10.3390/life15071012

**Published:** 2025-06-25

**Authors:** Aikaterini Agrafiotou, Evdoxia Sapountzi, Angeliki Margoni, Lampros Fotis

**Affiliations:** 1 Division of Pediatric Rheumatology, Department of Pediatrics, ATTIKON General Hospital, National and Kapodistrian University of Athens, 12462 Athens, Greece; cagraf@med.uoa.gr; 2 Outpatient Rheumatology Unit, 2nd Department of Pediatrics, Faculty of Health Sciences, School of Medicine, Aristotle University of Thessaloniki, AHEPA University General Hospital, 54636 Thessaloniki, Greece; sevdoxia@auth.gr; 3 Department of Biological Chemistry, Medical School, National and Kapodistrian University of Athens, 12462 Athens, Greece; mmargoni@med.uoa.gr

**Keywords:** biomarkers, immunity, adaptive, immunity, innate, immunophenotyping, intravenous immunoglobulins, Kawasaki disease

## Abstract

Kawasaki disease (KD) is a systematic inflammatory condition that results in vasculitis and possible progression to the development of coronary artery lesions if left untreated. Disease pathogenesis is not fully understood, and diagnosis is based on clinical symptoms, with limited reliability considering that KD progression is time sensitive. This is further complicated by the shared clinical characteristics with other febrile diseases. Early diagnosis and prompt treatment start are associated with good prognosis in most patients. However, up to 20% of patients are resistant to available therapeutic agents and would benefit from alternative regimens. Therefore, identification of biomarkers that can provide insights on disease pathogenesis are necessary to enable early diagnosis and initiation of treatment, as well as to predict treatment responses. To this end, immunophenotyping, most commonly by flow cytometry, has been crucial in identifying central factors in KD pathogenesis. The available literature on such factors is vast and may include contradictory findings. Therefore, we aimed to summarize the available literature of the last decade on the immunophenotype of KD, focusing on biomarkers associated with disease pathogenesis and those associated with treatment response. Our review highlights the role of cells of both the innate and adaptive immune system in disease pathogenesis, as well as the role of various secreted and cell surface proteins, including inflammatory cytokines, chemokines, complement receptors, and chemoattractants both in KD pathogenesis and in treatment response.

## 1. Introduction

Kawasaki disease (KD) is a pediatric inflammatory disease affecting the blood vessels. Its incidence varies greatly worldwide, with the highest reported in Japan (1 in 100 children <5 years) and the lowest in sub-Saharan Africa. In Europe and the US, the incidence ranges from 5–18 per 100,000 children <5 years [1]. Males are more affected than females and have a higher risk for complications [2]. The clinical characteristics of KD include fever, rash, mucocutaneous manifestations, lymphadenopathy, and elevated inflammatory parameters. Unfortunately, these features are common among several febrile illnesses in pediatric populations (e.g., measles, adenoviral infection, scarlet fever, dengue fever), thus complicating KD diagnosis [3].

The etiopathogenesis of KD remains unknown, although several factors have been considered since the first report of KD cases by Kawasaki in 1967 [4]. Both epidemiologic and clinical features strongly support an infectious etiology, possibly of an air-borne nature. The striking decline in KD cases globally during the COVID-19 pandemic, attributed to masking, social distancing, and school closures, further supports person-to-person spread via the respiratory route as a causative agent [5]. Further, there is broad consensus on the involvement of genetic factors in KD susceptibility [6], with variants in ITPKC, CASP3, and FCGR2a genes validated in independent cohorts across racial and ethnic groups [5,6]. More recently, KD was proposed to represent a syndrome with multiple etiological agents rather than a single disease entity based on immune profiling studies [5]. Finally, environmental factors, such as ozone, might also contribute to the occurrence of KD [7]. The interplay of all these factors leads to hyperactivation of the immune system, which ultimately contributes to vascular inflammation and damage. Generally, KD has a good prognosis, achieved with prompt treatment; however, coronary artery lesions (CALs), a severe complication of KD, may have fatal outcomes if left untreated [8].

In the context of immune system hyperactivation, immunophenotyping has emerged as a pivotal tool for characterizing the cellular and molecular profiles associated with the disease and for aiding in diagnosis. Immunophenotyping—the detailed analysis of cellular and protein markers [9]—has provided critical insights into the roles of various immune and non-immune cells in KD. These include neutrophils, monocytes, T cells, B cells, endothelial cells, and platelets, each uniquely contributing to vascular inflammation and remodeling in KD [10]. These immune profiles are closely associated with the response to standard treatments, including intravenous immunoglobulin (IVIG) and corticosteroids, as well as that to newly developed therapies, including anti-cytokine biologics. Therefore, the utility of immunophenotyping extends beyond pathogenesis, offering valuable insights into treatment response. This is critical, considering that up to 20% of patients are resistant to IVIG [11], complicated by the presence of genetic polymorphisms [12], highlighting the need to develop markers for patient selection. To this end, immunophenotyping has helped identify biomarkers such as elevated interleukin-6 (IL-6) and tumor necrosis factor α (TNF-α) levels, which are associated with IVIG resistance [13] and may serve as targets for adjunctive therapies.

This comprehensive narrative review explores KD’s immunophenotype in relation to its pathogenesis and treatment response, highlighting recent advances in our understanding of this enigmatic disease.

## 2. Literature Search

We performed a literature search in PUBMED, CENTRAL, Google Scholar, and Science Direct for articles in English published between 31 January 2014 and 31 January 2025 using the following search terms or their variants: Kawasaki disease, immunophenotype, innate immunity, adaptive immunity, cytokines, chemokines, biomarkers, pathogenesis, treatment response, IVIG, biologics. Most study types were included apart from case reports, editorials, and letters to the editor. Articles were initially selected based on the presence of the keywords in the title and/or abstract. The selected articles were then screened for content relevant to the topic of the review. Studies reporting findings on cell-surface proteins and proteins secreted from immune cells were included since these are mainly tested by immunophenotyping methods. Studies were excluded if they reported findings on other cell types or other proteins involved in KD, gene polymorphisms, or gene expression analyses (as immunophenotyping does not test for these). Animal studies and in vitro studies were also excluded.

Information on immunophenotyping relevant to KD pathogenesis and prediction of treatment response was extracted manually from the selected publications. If pertinent information was detected in a review article, the source article was used to extract further details. Since this was a narrative review, no statistical analysis was performed, and the extracted data were synthesized in a qualitative/narrative manner.

## 3. Immunophenotyping Kawasaki Disease: Insights for Disease Pathogenesis

Although KD pathogenesis is complex and governed by many factors, immunophenotyping studies have helped elucidate key mechanisms involved in the acute and subacute phases of KD.

### 3.1. Cellular Mechanisms

#### 3.1.1. Innate Immunity

The innate immune system, including neutrophils, monocytes, macrophages, and dendritic cells, is heavily implicated in the early stages of KD, with higher proportions of all innate cell types in patients than in healthy subjects [14], and is also critical for the development of coronary vasculitis [15].

Acute KD is characterized by infiltration of neutrophils in coronary arteries, contributing to the damage of the blood vessels through the release of reactive oxygen species (ROS) and proteolytic enzymes. Children with KD have higher levels of the brain natriuretic peptide, an essential modulator of neutrophil activation that regulates ROS production [16]. The rate of neutrophil activation is higher in KD and in KD complicated by CALs [16], while the neutrophil-to-lymphocyte (NLR) ratio has been demonstrated as a predictor of CAL formation [17].

Another characteristic of acute KD is the increased infiltration of circulating monocytes and macrophages into coronary arteries, which is associated with cytokine production, thus promoting inflammation. The increased number of CD14^+^ monocytes in KD patients with coronary artery abnormalities (CAAs) was previously suggested to serve as a marker of KD severity [18].

Earlier studies have reported contradictory findings for dendritic cells (DCs) in KD, with some showing fewer circulating myeloid DCs (mDCs) and/or plasmacytoid DCs (pDCs) in acute KD and other showing more circulating mDCs but not pDCs [19,20,21]. Moreover, one study showed significantly more mDCs in the CALs of patients with KD than in controls [22]. In contrast, a more recent study found no differences in DC subsets between patients with CALs and those without [21]. Such conflicting findings may result from the different markers used for classifying DCs. Regardless, they suggest abnormal numbers of DCs in patients with KD and a potential shift of circulating DCs toward the affected arteries where they may enhance T cell activation, thus promoting coronary arteritis in KD.

A characteristic finding of the subacute phase of KD is thrombocytosis, as indicated by increased platelet counts (>500,000/mm^3^) [23]. The occurrence of thrombocytosis is positively correlated with poor outcomes in patients with KD [24]. A higher platelet count was associated with longer fever duration and the length of hospital admission [24]. Moreover, the rate of platelet–neutrophil aggregate formation was significantly higher in patients with KD than in those with bacterial infection and normal volunteers, as well as in patients with CAA than in those without [25]. The authors also reported non-significantly higher rates shortly after IVIG administration and in IVIG non-responders [25]. Monocyte–platelet aggregates were also higher in KD than in febrile and healthy control samples, remaining high even 3 months after KD diagnosis, suggesting that activated platelets remain long after inflammation has decreased [26].

#### 3.1.2. Adaptive Immunity

Cells of the adaptive immune system are also affected in acute KD. CD8 T cells have been reported to decrease in the circulation but aggregate in coronary arteries [27,28]. Increased numbers have also been shown for CD69^+^ CD8 T cells [29], T helper cell type 1 (Th1) and Th2 cells [30], as well as Th17 cells [31]. In contrast, decreased numbers of T regulatory cells (Tregs) were reported in acute KD [31]. Consistently, retinoic acid receptor-related orphan receptor gt (RORgt), a Th17 transcription factor, was found to be significantly upregulated [31,32], whereas Treg factors such as forkhead box P3 (FoxP3), glucocorticoid-induced TNF receptor family-related protein (GITR), and cytotoxic T-lymphocyte associated protein 4 (CTLA-4) were significantly decreased in acute KD [31,33]. The increased number of Th17 cells was associated with the induction of inflammation, a characteristic of KD, further enhanced by Treg downregulation [32]. Guo et al. found that while patients with KD had higher levels of Th17 cells, they had lower percentages of CD4^+^ T cells [34]. In agreement, fewer peripheral CD4^+^ T cells were detected in patients with KD than in healthy subjects [21]. Oppositely, Ding et al. reported more CD4^+^ T cells in KD than in healthy controls but found fewer circulating CD3 and CD8 T cells in KD than in febrile and healthy controls, as well as fewer CD16 and CD56 cells (natural killer (NK) cells) in both the KD and febrile groups than in the healthy control group [35]. The authors also demonstrated that different lymphocyte subsets could discriminate between complete and incomplete KD [35].

Increased numbers of B cells (CD19^+^) have also been reported in KD [35]. This increase might be driven by a specific B cell subset, CD19^+^CD27^high^ peripheral blood antibody-secreting B cells, suggesting its role in KD development [36]. Variations in subsets of antibody-secreting cells and memory B cells were also associated with KD pathogenesis, although the exact contribution of these cells is not known as variable results were obtained in terms of their correlation with laboratory findings [37].

Circulating follicular helper T (cTfh) cells have also been implicated in KD pathogenesis [38,39]. Although the total number of these cells does not seem to differ between control and acute KD or KD in remission, Xu et al. reported variations in the percentage of specific cell subsets. Specifically, the percentages of ICOS^high^PD-1^high^, ICOS^+^PD-1^+^, ICOS^−^PD-1^+^, and CD45RA^−^IL-21^+^ cTfh cells were significantly elevated in acute KD, which was considered to reflect the activation of cTfh cells, maintained in the remission phase [38]. In a subsequent study, the authors showed altered numbers of two other cTfh subsets (more CXCR3^+^ CCR6^−^ and fewer CXCR3^−^CCR6^−^ cells) in KD and in KD with CALs [39]. The involvement of these different subsets of cTfh cells in KD may be mediated by their secreted cytokines.

Collectively, the abnormal composition of the adaptive immune system promotes inflammation, which results in the vasculitis observed in patients with KD [34,40].

### 3.2. Secreted Proteins

The identified proteins secreted by immune cells that showed significant changes in KD or after treatment are listed in Table 1.

#### 3.2.1. Inflammatory Cytokines

KD is characterized by significantly increased cytokine and chemokine profiles, primarily due to changes in altered immune cell composition [88].

IL-6 emerges as an important player in KD pathogenesis, with levels at least 20 times higher than in healthy controls and about 5 times higher in the acute versus the subacute phase of KD [44,45]. IL-6 levels differentiate incomplete from complete KD [43] and are markedly elevated in patients with Kawasaki disease shock syndrome (KDSS) and those with CALs [30,46]. IL-6-mediated damage involves megakaryocyte maturation, causing thrombocytosis, and polyclonal B cell autoantibody production, leading to endothelial damage and vasculitis [88].

TNF-α is also significantly elevated in acute KD [89]. Through its soluble receptors, TNFR1 and TNFR2, TNF-α upregulates metalloproteinase 9, causing elastin breakdown and aneurysms in the vascular walls [90]. This has been inferred to be one of the contributing mechanisms of CAL formation in KD, supported by significantly higher levels of TNF-α in KD patients with CAL [91]. However, TNF-α has a pleiotropic effect in KD, being involved in leukocyte recruitment to target sites, modulation of other cytokines, as well as regulation of cell death [92]. TNFR1 and TNFR2 levels were shown to be significantly decreased after treatment with plasma exchange [51].

Other pro-inflammatory cytokines with significantly higher levels in patients with KD include IL-1b (produced mainly by macrophages but also neutrophils, epithelial cells, and endothelial cells), IL-17 (Th17 cells), IL-10 (subsets of immune cells, including CD4^+^ T cells), IL-18 (monocytes, macrophages), IL-27 (activated antigen-presenting cells), IL-31 (Th2 cells), interferon gamma (IFN-γ) (activated T cells, NK cells), and IL-33 (damaged epithelial cells, macrophages, DCs) (Table 1).

Several cytokines correlate with KD complications, including CALs, KDSS, and macrophage activation syndrome (MAS). IL-6 levels > 66.7 pg/mL, IL-10 > 20.85 pg/mL, and IFN-γ > 8.35 pg/mL increase KDSS risk [46]. Both IL-17 and IL-31 are highly elevated in patients with CALs, with IL-17 positively correlating with the coronary artery z-score [55,56]. IL-18 intensifies coronary arteritis in KD and serves as the best predictor for CAL formation compared to other biomarkers such as IL-17 and TNF-α [57,93]. Both IL-10 and IFN-γ are increased in patients with KDSS [46], while IL-18 is significantly elevated in KD complicated with MAS [58,94].

Anti-inflammatory cytokines show contradictory findings. IL-35 levels have been reported as both decreased in patients with KD and in those who develop CALs compared with febrile or healthy controls [62] and as increased [26], possibly due to methodological differences. IL-35 is produced by CD4^+^ Treg cells, activated DCs, macrophages, endothelial cells, and aortic smooth muscle cells, and is suggested to exert immunosuppressive effects and to decrease the risk of progression of inflammatory and autoimmune diseases. Despite the conflicting results, both studies suggested that IL-35 has protective effects against inflammatory processes and CAL formation. IL-37 levels are decreased in the serum of patients with KD, further supporting a protective role of anti-inflammatory cytokines in KD progression, also indicated by a series of in vitro experiments [63].

#### 3.2.2. Chemokines and Cell Adhesion Molecules

Chemokines and cell adhesion molecules mediate the infiltration of blood vessels by immune cells, a crucial aspect of KD pathogenesis but also of other vascular diseases. The levels of several chemokines, including CXCL9, CXCL10, CCL17, CCL11 (eotaxin), and monocyte chemoattractant protein-1 (MCP-1) are increased in acute KD, while those of CXCL9, CXCL10, and CCL17 are also higher in patients who develop CALs [72,73].

The circulating levels of the cell adhesion molecules semaphorin 7A and semaphorin 4D are also significantly elevated in KD, with semaphorin 4D also being elevated in patients with CALs [75,76]. As a transmembrane protein, semaphorin 7A is expressed on activated T cells, while semaphorin 4D is expressed on various immune cell types, including T cells, B cells, neutrophils, monocytes/macrophages, and platelets. When these molecules are cleaved from the cell surface, their soluble forms can activate the respective receptors expressed on different cell types. Semaphorin 4D exerts pro-inflammatory effects via its receptor plexin B in various inflammatory diseases. In KD, it is cleaved by ADAM17 specifically from the surface of neutrophils, thus inducing the secretion of pro-inflammatory cytokines from endothelial cells [76], suggesting a role in KD pathogenesis and CAL formation.

#### 3.2.3. Complement Factors

The damage to endothelial cells in KD results in systemic vasculitis or aneurysms in coronary arteries. Complement factors produced by vascular endothelial cells are important mediators of the inflammatory response, and the levels of several complement factors are lower in patients with KD than in healthy controls, possibly contributing to the dysregulated immune response in these patients [70,71]. Our literature search showed that the complement receptor CD11b, expressed in monocyte/macrophages, granulocytes, and NK cells, is significantly decreased in KD. Although no differences were noted in CD59 levels, expressed on monocytes (among other cell types), increased levels were detected in the acute versus the subacute phase of KD [70]. This finding warrants further investigation as the role of CD59 is to prevent the formation of the membrane attack complex of the complement system [95], and hence higher levels would be expected in the subacute rather than in the acute phase of KD.

#### 3.2.4. Other

C-reactive protein (CRP) is an acute-phase reactant protein released by peripheral blood mononuclear cells as a result of inflammation. Its secretion is induced by IL-6 and its effect maintained by IL-1 [96]. CRP levels are significantly elevated in KD (versus febrile controls) [97] and in patients with CALs [79,98]. However, the mechanism underlying CRP involvement in KD pathogenesis and CAL development remains elusive.

CD84, expressed on T and B cells but also on monocytes/macrophages, granulocytes, DCs, and mast cells, is robustly expressed on inflammatory cells in the arterial walls of patients with KD [78].

The activating Fcγ receptors (FcγR) I, III, and IIa, expressed on various immune cell types, are highly expressed in KD versus controls. FcγRI (CD64) expression is specifically increased on neutrophils and monocytes at the onset of KD flare-ups, indicating a possible role in KD pathogenesis [99]. In contrast, the inhibitory FcγRIIb is lower in patients with KD and in those who develop CALs [80], suggesting a protective role in these patients.

Chitinase-3-like protein 1, also known as YKL-40, is a glycoprotein secreted from activated neutrophils and macrophages in different tissues as a result of inflammation. YLK-40 levels are elevated in KD, specifically in the acute phase, and remain high in the subacute phase [81], suggesting that YLK-40 may be a useful marker of KD activity.

Leukocyte-associated Ig-like receptor-1 (LAIR-1) was found to be increased on neutrophils but decreased on CD4^+^ and CD8^+^ T lymphocytes of patients with KD, with high neutrophil expression also in patients with CALs. Soluble LAIR-1 levels are also elevated in the KD and KD+CAL [83]. These findings suggest that LAIR-1 might be implicated in KD pathogenesis and CAL formation, while its soluble form might be a useful biomarker. Activin type IIA receptor (ActRIIA) is increased on the surface of CD8^+^ T cells, CD19^+^ B cells, and CD14^+^ monocytes in the acute phase of KD, whereas the serum levels of its ligand, activin A, are decreased [86,100]. Activin A is synthesized and secreted by various immune cells such as T cells, B cells, monocytes, dendritic cells, and mast cells and inhibits ActRIIA expression on monocytes in KD [100]. Collectively, these findings suggest the overactivation of the above cell types in KD, contributing to disease pathogenesis, and further propose a possible protective role of activin A, which can be exploited when considering new treatments for KD.

Circulating neutrophils secrete S100A12 in the early stages of KD. Accordingly, serum levels of S100A12, as well as those of related molecules S100A8 and A8, were found to be elevated in acute KD, and in patients with CALs. S100A12 activates monocytes and triggers IL-1b production, in turn activating endothelial cells of the coronary artery and contributing to KD pathogenesis [84].

The levels of platelet-activating factor (PAF) are significantly higher in KD than in healthy or febrile controls. PAF is a potent pro-inflammatory molecule produced by various cell types, including macrophages, monocytes, and neutrophils, and can activate endothelial cells, neutrophils, and monocytes, leading to their adherence and migration. Moreover, activation of PAF receptors on monocytes leads to increased secretion of MCP-1 and TNF-α [101]. These results suggest a role of PAF in KD pathogenesis, and PAF serum levels > 225.52 ng/mL serve as a significant risk factor for CAL formation [85].

**Figure 1:**
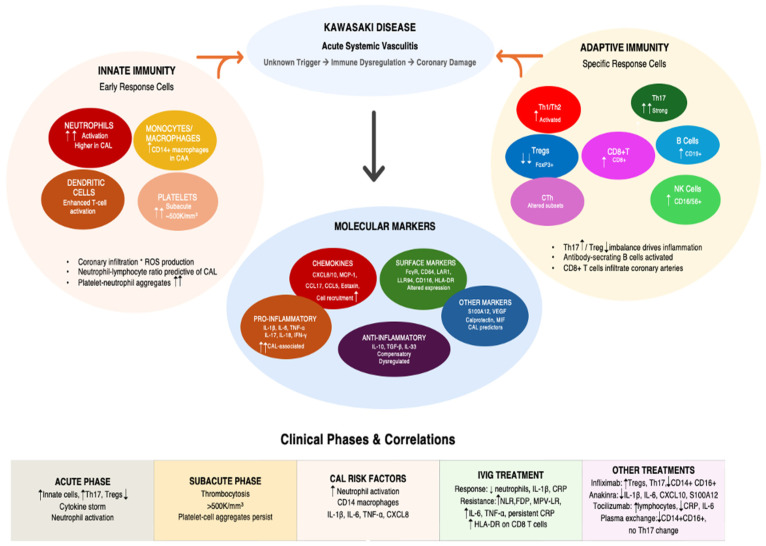
Kawasaki disease—immunological mechanisms and clinical correlations. The diagram illustrates the immunological mechanisms underlying Kawasaki disease, showing the interplay between innate immunity, adaptive immunity, and molecular markers. The bottom section displays clinical phases and treatment correlations. Arrows indicate the pathophysiological progression from immune activation to molecular marker expression and clinical manifestations. Abbreviations: ↑, increase; ↑↑, marked increase; ↓, decrease; ↓↓, marked decrease; CAA, coronary artery aneurysm; CAL, coronary artery lesion; CCL, C-C motif chemokine ligand; CD, cluster of differentiation; CRP, C-reactive protein; CTh, circulating T helper cells; CXCL, C-X-C motif chemokine ligand; FDP, fibrin degradation products; FcγR, Fc gamma receptor; FoxP3, forkhead box P3; HLA-DR, human leukocyte antigen DR isotype; IFN-γ, interferon-gamma; IL, interleukin; IVIG, intravenous immunoglobulin; LAR1, leukocyte-associated receptor 1; LLR94, leukocyte immunoglobulin-like receptor 94; MCP-1, monocyte chemoattractant protein-1; MIF, macrophage migration inhibitory factor; MPVLR, mean platelet volume-to-lymphocyte ratio; NK, natural killer; NLR, neutrophil-to-lymphocyte ratio; ROS, reactive oxygen species; S100A12, S100 calcium-binding protein A12; TGF-β, transforming growth factor-beta; Th, T helper; TNF-α, tumor necrosis factor-alpha; Tregs, regulatory T cells; VEGF, vascular endothelial growth factor.

Collectively, these immunophenotyping findings demonstrate the complex interplay of innate and adaptive immune dysregulation in KD pathogenesis (Figure 1).

## 4. Immunophenotype and Response to Treatment

### 4.1. Response to IVIG Treatment

The standard treatment of KD consists of IVIG combined with high-dose aspirin [102]. This regimen decreases fever and inflammation and reduces the risk of CAL development [103]. However, some patients develop IVIG resistance, prompting adjunctive therapies including corticosteroids [103]. The cause of IVIG resistance remains unclear, although certain clinical features and laboratory parameters, as well as gene polymorphisms, have been implicated in the process [12]. The mechanism underlying IVIG efficacy involves changes in the immune cell repertoire, thus affecting cytokine and chemokine profiles [20].

#### 4.1.1. Cellular Changes

Neutrophils and neutrophil lineage cells are massively reduced in the subacute phase after IVIG treatment, with >90% reduction in IL-1β–expressing circulating neutrophils and significant decreases in neutrophil-produced IL-1β levels, but no significant effect on other IL-1β–expressing populations [41]. IVIG seems to target specifically the mature IL-1β–producing neutrophils rather than the neutrophil progenitors [41]. In contrast, IVIG-resistance is associated with higher neutrophil percentage, NLR, platelet-to-lymphocyte ratio (PLR), and mean platelet volume-to-lymphocyte ratio (MPVLR) [104].

Monocytes represent a major target of IVIG therapy [105]. Elevated CD14^+^ CD16^+^ “intermediate” monocytes in acute KD are significantly reduced after IVIG treatment [64]. Notably, this monocyte subpopulation is significantly lower in IVIG-resistant than in IVIG-responsive patients before treatment [106].

T lymphocytes show complex responses. CD69^+^ CD8 T cells serve as markers of both disease progression and IVIG response [29]. The number of CD4 T cells expressing human leukocyte antigen-DR isotype (HLA-DR) increases significantly after IVIG in both responsive and resistant patients, whereas that of HLA-DR-positive CD8 T cells increase only in IVIG-resistant patients [64]. Different lymphocyte subsets are able to discriminate between IVIG-responsive and non-responsive patients [35]. Combination of IVIG with corticosteroids leads to a more efficient increase in CD3^+^, CD4^+^, and CD4^+^/CD8^+^ T cell subsets (*p* < 0.05), and a more efficient decrease in CD8^+^ T cells [107].

Other immune cells: IVIG infusion increases the frequency of Tregs and the activation of the immunoregulatory CD56^high^ NK cells and CD56^+^ T cells, whereas it reduces the frequency of CD107a-positive, CD56^low^ cytotoxic NK cells. Moreover, it increases the proportion of CD56^high^ NK cells expressing the activating receptor CD336 [108]. Reduced numbers of Th17 cells are observed in KD patients treated with IVIG plus aspirin [32].

#### 4.1.2. Cytokine and Biomarker Changes

IL-6 levels normalize following IVIG treatment in responsive patients, correlating with a concomitant reduction in CRP [45,97]. In contrast, they remain significantly higher in IVIG-resistant patients, while persistently high levels post-IVIG are also associated with CAL formation [109].

Other cytokines such as IL-2, IL-4, IL-2, IL-10, TNF-α, and IFN-γ show conflicting results. No significant differences between IVIG-responsive and resistant patients were found by Kong et al. [109], increased TNF-α levels were detected in unresponsive patients by Hu et al. [65], while significantly higher IFN-γ and TNF-α levels in IVIG-responsive patients were reported by Zhang et al. [110]. Higher levels of IFN-γ were also confirmed as an independent predictor of IVIG-resistance [48]. In responsive patients, IVIG significantly reduces IL-10 levels, with greater improvement noted in older children [111].

Additional biomarkers: High pre-treatment CRP levels (>100 mg/L) and elevated CRP-to-albumin ratios independently predict IVIG resistance [11,112], while high CRP levels pre and post-treatment are also associated with non-responsiveness [47]. Increased T cell HLA-DR expression is associated with IVIG resistance [82], while elevated CD64 (FcγRI) decrease significantly after IVIG [99]. IVIG therapy is efficient in restoring the levels of abnormally expressed chemokines in KD patients (Table 1).

### 4.2. Response to Other Treatments

The elevated levels of inflammatory cytokines have prompted the discovery of targeted therapies to overcome cases of IVIG resistance in patients with KD. Such anti-cytokine therapies include the TNF inhibitors infliximab and etanercept, the IL-6 receptor inhibitor tocilizumab, and the IL-1 receptor antagonist anakinra, all of which have shown promising results in IVIG-refractory KD [20,113,114,115]. A previous systematic review and meta-analysis reported reduced frequency of treatment resistance when using anti-cytokine biologics, although these did not seem to be as effective in reducing the risk for CAL formation in KD patients [116]. Severe KD that is resistant to IVIG may also benefit from plasma exchange therapy [117].

Our review revealed a limited number of studies for these therapies. Infliximab increases Treg frequency and Th17 cells in KD patients [118], whereas plasma exchange does not alter Th17 cell numbers [119]. Both infliximab and plasma exchange significantly reduce the numbers of CD14^+^ CD16^+^ monocytes [118,119]. Anakinra treatment decreases the levels of most serum inflammatory markers in IVIG-resistant patients, with changes being more pronounced for IL-6, IL-10, CXCL10, and S100A12 [115]. Further studies are needed to identify how these treatments may affect KD immunophenotype and the mechanisms underlying their positive effects for patients.

## 5. Clinical Applications

### 5.1. Diagnosis

The immunophenotype markers identified in this review provide objective, quantifiable parameters that reflect the underlying immune dysregulation in KD, including elevated neutrophil-to-lymphocyte ratios, increased inflammatory cytokines (including among others IL-6, TNF-α, IL-1β, IL-17), altered T cell subsets (increased Th17, decreased Tregs), activated monocyte populations (CD14^+^CD16^+^), and platelet activation markers. The integration of these immunophenotyping findings with advanced diagnostic approaches offers significant potential for improving KD diagnosis in clinical practice. Traditional diagnostic criteria rely on subjective clinical manifestations that can be challenging to identify, particularly in incomplete KD cases or within the critical first 5 days of fever onset [3]. Recent machine learning advances have demonstrated exceptional diagnostic performance, with XGBoost algorithms achieving AUC values of 0.9833–0.9999 and sensitivity of 93.85–99.82% for distinguishing KD from other febrile illnesses using routine laboratory parameters [120]. Therefore, integration of immunophenotyping markers with machine learning frameworks could enhance diagnostic accuracy across diverse populations while reducing dependence on subjective clinical assessments.

### 5.2. Prediction of IVIG Resistance

Predicting IVIG resistance remains crucial for optimizing KD treatment strategies, as resistant patients face significantly higher risks of coronary artery complications. To this end, several scoring systems have been developed using readily available laboratory and clinical parameters, demonstrating good predictive accuracy in Japanese populations [121,122,123]. However, validation studies have consistently shown poor performance outside Japan. A 2023 meta-analysis of 48 studies from diverse populations globally found that all five major scoring systems (Kobayashi, Egami, Sano, Formosa, Harada) showed disappointing predictive ability, with low positive predictive values (0.14–0.39) and high negative predictive values (0.85–0.92) [124]. European and North American validation studies reported sensitivities as low as 14% and 33%, respectively, with these scores missing up to 86% of truly IVIG-resistant patients [125,126]. Recognition of these limitations has prompted integration of immunophenotype markers such as NLR and PLR, which have shown superior performance compared to traditional scores (OR 5.34) [127]. Moreover, recent machine learning advances offer promising alternatives to traditional scoring systems for IVIG resistance prediction. A review of 21 AI studies found that machine learning models demonstrated superior accuracy compared to traditional scores [128]. However, they are limited by predominantly relying on retrospective data, class imbalance issues, and limited validation across diverse populations (90% of studies were conducted in Asian hospitals).

The immunophenotype markers highlighted in this review provide mechanistic insights into treatment resistance that could enhance predictive models. We found that IVIG-resistant patients demonstrate distinct immunological profiles including higher neutrophil percentages, elevated NLR and PLR, persistently elevated IL-6 and TNF-α levels, increased CRP (>100 mg/L), higher CRP-to-albumin ratios, and altered T cell activation patterns with increased HLA-DR expression. Incorporation of these profiles to machine learning frameworks represents a promising approach for developing population-independent prediction tools that could enable personalized treatment strategies, including early escalation to anti-cytokine therapies (infliximab, anakinra, tocilizumab) for high-risk patients, ultimately reducing coronary complications and improving long-term outcomes.

## 6. Conclusions

We aimed to summarize the immunophenotype of KD focusing on pathogenesis and treatment response. Our review highlights the hyper-activation of the immune system, with altered frequencies of both innate and adaptive immune cells, elevated levels of pro-inflammatory serum cytokines such as IL-6 and TNF-α, and reduced levels of anti-inflammatory cytokines such as IL-35 and Il-37, as well as the role of several other inflammatory molecules in KD pathogenesis. Although IVIG treatment is shown to restore many of these changes, the abnormal levels of certain cells and factors persist in IVIG-resistant patients. Therapy with other agents, including anti-cytokine biologics and corticosteroids, seems to be beneficial in limited aspects of KD pathophysiology but may aid in reducing IVIG resistance. The integration of immunophenotyping markers with machine learning approaches represents a promising avenue for KD clinical management, overcoming population-specific limitations of current scoring systems and enabling personalized treatment strategies, thus ultimately improving diagnostic precision and reducing coronary artery complications.

## Figures and Tables

**Table 1:** Serum biomarkers for KD pathogenesis and treatment response.

Marker	Role in KD		Change at Specific KD Phase or After Treatment ^a^	References
	Pathogenesis	Treatment Response		
Cytokines				
IL-1β	Y	Y	Serum levels: KD + CALs >> KD no CALs >> febrile and healthy controls ↓↓ after IVIG	[41,42]
IL-2 receptor	Y	Y	Serum levels: KD >> controls or reference range ↓↓ after IVIG	[43]
IL-6	Y	Y	Serum levels ↑ in KD, ↑ in acute KD KD + CALs >> KD no CALs	[33,34,42,44,45,46,47,48,49,50]
			↓↓ after IVIG, plasma exchange, infliximab, or anakinra	[30,33,45,47,50,51,52]
IL-8	Y	-	↑ in acute KD, KD >> MIS-C >> healthy controls	[53]
IL-10	Y	Y	↑ in KD, ↑ in IVIG-resistant KD patients. ↓↓ after IVIG or anakinra	[30,42,48,50]
IL-17	Y	Y	KD >> febrile and healthy controls	[34,42,54,55,56]
			↓↓ after plasma exchange and gradually after IVIG	[34,54]
IL-18	Y	Y	KD >> healthy controls Associated with CAL.	[57,58]
			↓↓ after IVIG	[59]
IL-23	Y	-	KD + CALs >> KD no CALs >> infectious disease and healthy controls	[31]
IL-27	Y	-	Serum levels: KD + CALs >> KD no CALs >> healthy controls	[42]
IL-31	Y	Y	Serum levels KD >> febrile and healthy controls Significantly associated with CALs	[34,55]
			↑↑ after IVIG	[55]
IL-33	Y	Y	Acute KD >> healthy controls; KD << febrile controls ↓↓ after IVIG	[60,61]
IL-35	Y	-	KD << febrile and healthy controls KD + CAL << no CAL	[62]
			KD >> healthy controls	[26]
IL-37	Y	-	KD << febrile and healthy controls	[63]
IFN-γ	Y	Y	KD + KDSS >> KD >> MIS-C >> healthy controls ↓↓ after IVIG	[30,46,53]
TNF-α	Y	Y	Acute KD >> healthy controls ↑↑ KD + CALs	[30,58,64]
			↓↓ after plasma exchange and infliximab treatment. ↓↓ after IVIG in KD patients without CALs and in IVIG responders ↑ after IVIG in KD patients with CALs and in IVIG-resistant patients; predictor of IVIG-resistance	[30,48,51,52,65]
Soluble TNFR1 and TNFR2	Y	Y	Acute >> subacute KD TNFR2 and TNFR1/2 ratio: KD + CALs >> no CALs ↓↓ after plasma exchange and infliximab treatment TNFR1 remains high in infliximab-resistant patients.	[51,52,66,67]
G-CSF	Y	Y	KD >> MIS-C >> healthy controls ↓↓ after plasma exchange, infliximab, and IVIG treatment Remains high in infliximab-resistant patients	[41,51,52,53]
sCD40L	Y	-	KD >> febrile controls	[61]
IP-10	Y	Y	↑ in KD ↓ after infliximab treatment	[52]
TGF-β	Y	-	↑ in acute KD >> infectious disease and healthy controls	[31]
Complement receptors				
CD11b	Y	Y	Mean CD11b ↓ in KD before and after IVIG	[68,69]
CD59	Y	-	subacute KD << acute KD KD + CAL << no CAL	[70,71]
Chemokines and cell adhesion molecules				
CXCL9 and CXCL10	Y	Y	Both ↑ in acute KD; CXCL9: KD + CAL >> no CAL ↓↓ after IVIG CXCL10: ↓↓ after anakinra treatment	[50,61,72,73]
MCP-1 (CCL2)	Y	Y	↑ in acute KD ↓↓ after IVIG	[73]
CCL5	Y	-	↑ in acute KD, KD >> healthy controls	[53]
Eotaxin (CCL11)	Y	Y	↑ in acute KD ↓↓ after IVIG	[73]
CCL17	Y	Y	KD >> healthy controls; KD + CAL >> no CAL	[73]
			↓↓ after IVIG	[74]
Semaphorin 7A	Y	-	Serum levels ↑ in KD	[75]
Semaphorin 4D	Y	-	Serum levels ↑ in acute KD and in patients with CALs ↓ in convalescent phase	[76]
P-Selectin (CD62P)	Y	-	2~3-fold higher expression in KD platelets than in healthy platelets	[77]
Other				
VEGF (angiogenic factor)	Y	-	KD + CALs >> no CALs	[78]
CD84 (Signaling lymphocyte activation molecule)	Y	-	Robust expression in inflammatory cells in arterial walls in 6/7 acute and 4/5 chronic cases.	[78]
CRP	Y	Y	↑ in KD + CAL Level >100 mg/L at diagnosis is an independent risk factor of IVIG resistance.	[11,79]
Fcγ receptors	Y	Y	FcγRIII and FcγRIIa levels: KD >> controls	[80]
			FcγRIIb: KD << controls; KD + CALs << no CALs	[80]
			FcγRI (CD64): ↑↑ expression on neutrophils and monocytes at the onset of KD flare-ups. ↓↓ after IVIG	[81]
LILRs/ILTs (receptors involved in immune regulation)	Y	Y	LILRB4 (ILT3/LIR-5/CD85k): ↑ in acute KD, expressed uniquely on antibody-secreting B cells; ↓ after IVIG LILRB1 (ILT2/CD85j): ↑ in acute KD and after IVIG in naïve and memory B cells, antibody-secreting cells, and monocytes.	[36]
HLA-DR (MHC molecule)	-	Y	↑ in IVIG-resistant KD patients.	[82]
LAIR-1 (receptor involved in immune regulation)	Y	-	Significantly increased in KD >> healthy controls; KD + CAL >> no CAL	[83]
YKL-40 (endothelial marker)	Y	-	Acute KD >> disease and healthy controls	[81]
S100A12 (calcium-binding protein)	Y	Y	↑↑ in acute KD KD + CAL >> no CAL ↓↓ after IVIG, no change in non-responsive patients ↓↓ after anakinra treatment	[50,84]
PAF (phospholipid mediator)	Y	-	Acute KD >> febrile and healthy controls KD + CAL >> no CAL	[85]
Activin receptor IIA	Y	-	Increased expression on CD8^+^ T cells and CD19^+^ B cells in KD.	[86]
Cathelicidin (LL-37) (anti-microbial peptide)		-	KD >> pneumonia and healthy controls	[87]

^a^ Only significant changes are shown. Abbreviations: ↑, increase; ↑↑, marked increase; ↓, decrease; ↓↓, marked decrease; CAL, coronary artery lesion; CCL, C-C motif chemokine ligand; CRP, C-reactive protein; CXCL, CXC motif chemokine ligand; Fc, fragment crystallizable; FcγR, Fc gamma receptor; G-CSF, granulocyte colony-stimulating factor; HLA-DR, human leukocyte antigen-DR; IFN-γ, interferon gamma; IL, interleukin; ILT, immunoglobulin-like transcript; IP-10, interferon gamma-induced protein 10; IVIG, intravenous immunoglobulin; KD, Kawasaki disease; KDSS, Kawasaki disease shock syndrome; LAIR, leukocyte-associated Ig-like receptor-1; LILR, leukocyte Ig-like receptor; MCP-1, monocyte chemoattractant protein-1; MHC, major histocompatibility complex; MIS-C, multisystem inflammatory syndrome in children PAF, platelet-activating factor; sCD40L, soluble CD40 ligand; S100A12, S100 calcium-binding protein A12; TGF-β, transforming growth factor beta; TNF-α, tumor necrosis factor alpha; TNFR, tumor necrosis factor receptor; VEGF, vascular endothelial growth factor; Y, yes.

## Abbreviations

The following abbreviations are used in this manuscript:

ADAM17	A disintegrin and metalloproteinase 17
ActRIIA	activin type IIA receptor
CAA	coronary artery abnormalities
CAL	coronary artery lesions
CCL	C-C motif ligand
CCR	C-C chemokine receptor
CRP	C-reactive protein
cTfh	circulating follicular helper T
CTLA-4	cytotoxic T-lymphocyte associated protein 4
CXCL	CXC chemokine ligand
CXCR	CXC chemokine receptor
DC	dendritic cell
FcγR	Fcγ receptor
FoxP3	forkhead box P3
GITR	glucocorticoid-induced TNF receptor family-related protein
HLA-DR	human leukocyte antigen-DR isotype
ICOS	inducible costimulatory
IFN-γ	interferon gamma
IL	interleukin
IVIG	intravenous immunoglobulin
KD	Kawasaki disease
KDSS	Kawasaki disease shock syndrome
LAIR-1	leukocyte-associated Ig-like receptor-1
MAS	macrophage activation syndrome
MCP-1	monocyte chemoattractant protein-1
mDC	myeloid dendritic cell
MPA	monocyte–platelet aggregates
NK	natural killer
PAF	platelet-activating factor
PD-1	programmed cell death protein 1
pDC	plasmacytoid dendritic cell
RORgt	retinoic acid receptor-related orphan receptor gt
ROS	reactive oxygen species
Th1/2	T helper cell type ½
TNF-α	tumor necrosis factor α
Treg	T regulatory cell

## References

[1] Elakabawi K., Lin J., Jiao F., Guo N., Yuan Z. (2020). Kawasaki disease: Global burden and genetic background. Cardiol. Res..

[2] Rowley A.H., Shulman S.T. (2018). The epidemiology and pathogenesis of Kawasaki disease. Front. Pediatr..

[3] Singh S., Jindal A.K., Pilania R.K. (2018). Diagnosis of Kawasaki disease. Int. J. Rheum. Dis..

[4] Kawasaki T., Kosaki F., Okawa S., Shigematsu I., Yanagawa H. (1974). A new infantile acute febrile mucocutaneous lymph node syndrome (MLNS) prevailing in Japan. Pediatrics.

[5] Burns J.C. (2024). The etiologies of Kawasaki disease. J. Clin. Investig..

[6] Sapountzi E., Kotanidou E.P., Tsinopoulou V.R., Kalinderi K., Fidani L., Giannopoulos A., Galli-Tsinopoulou A. (2024). Kawasaki disease: An update on genetics and pathophysiology. Genet. Test. Mol. Biomark..

[7] Shuhan H., Zhimeng H., Yaxuan L., Jingxuan F., Ruiqi C., Wenxing G., Huifen Z., Xiaoqing Y., Wu J., Lilin Z. (2025). Ozone exposure is positively correlated with the occurrence of Kawasaki disease in Chinese children. Pediatr. Res..

[8] Duarte R., Cisneros S., Fernandez G., Castellon D., Cattani C., A Melo C., Apocada A. (2010). Kawasaki disease: A review with emphasis on cardiovascular complications. Insights Imaging.

[9] Herold N.C., Mitra P. (2025). Immunophenotyping. StatPearls.

[10] Hara T., Yamamura K., Sakai Y. (2021). The up-to-date pathophysiology of Kawasaki disease. Clin. Transl. Immunol..

[11] Li W., Zhang L., Wang Z., He X., Lin H., Wang Y., Yuan J., Xie X., Zhang X., Qin Y. (2022). Predictors for intravenous immunoglobulin resistance in patients with Kawasaki disease. Int. J. Clin. Pract..

[12] Sapountzi E., Fidani L., Giannopoulos A., Galli-Tsinopoulou A. (2023). Association of genetic polymorphisms in Kawasaki disease with the response to immunoglobulin therapy. Pediatr. Cardiol..

[13] Sato S., Kawashima H., Kashiwagi Y., Hoshika A. (2013). Inflammatory cytokines as predictors of resistance to intravenous immunoglobulin therapy in Kawasaki disease patients. Int. J. Rheum. Dis..

[14] Xie Z., Huang Y., Li X., Lun Y., Li X., He Y., Wu S., Wang S., Sun J., Zhang J. (2022). Atlas of circulating immune cells in Kawasaki disease. Int. Immunopharmacol..

[15] Hara T., Nakashima Y., Sakai Y., Nishio H., Motomura Y., Yamasaki S. (2016). Kawasaki disease: A matter of innate immunity. Clin. Exp. Immunol..

[16] Hu J., Qian W., Yu Z., Xu T., Ju L., Hua Q., Wang Y., Ling J.J., Lv H. (2020). Increased neutrophil respiratory burst predicts the risk of coronary artery lesion in Kawasaki disease. Front. Pediatr..

[17] Sarejloo S., Shahri M.M., Azami P., Clark A., Hass E., Salimi M., Lucke-Wold B., Sadeghvand S., Khanzadeh S. (2022). Neutrophil to lymphocyte ratio as a biomarker for predicting the coronary artery abnormality in Kawasaki disease: A meta-analysis. Dis. Markers.

[18] Furukawa S., Matsubara T., Yabuta K. (1992). Mononuclear cell subsets and coronary artery lesions in Kawasaki disease. Arch. Dis. Child..

[19] Suda K., Kishimoto S., Takahashi T., Nishino H., Okamura H. (2013). Circulating myeloid dendritic cells is decreased in the acute phase of Kawasaki disease. Exp. Clin. Cardiol..

[20] Burns J., Song Y., Bujold M., Shimizu C., Kanegaye J., Tremoulet A., Franco A. (2013). Immune-monitoring in Kawasaki disease patients treated with infliximab and intravenous immunoglobulin. Clin. Exp. Immunol..

[21] Wang N., Chen Z., Zhang F., Zhang Q., Sun L., Lv H., Wang B., Shen J., Zhou X., Chen F. (2022). Intravenous immunoglobulin therapy restores the quantity and phenotype of circulating dendritic cells and CD4+ T cells in children with acute Kawasaki disease. Front. Immunol..

[22] Yilmaz A., Rowley A., Schulte D.J., Doherty T.M., Schröder N.W., Fishbein M.C., Kalelkar M., Cicha I., Schubert K., Daniel W.G. (2007). Activated myeloid dendritic cells accumulate and co-localize with CD3+ T cells in coronary artery lesions in patients with Kawasaki disease. Exp. Mol. Pathol..

[23] Kim B.J., Choi A., Kim S., Han J.W. (2024). The incidence of periungual desquamation and thrombocytosis in Kawasaki disease and the importance of systematic observation in the subacute phase. Front. Pediatr..

[24] Park J.H., Choi H.J. (2021). Clinical implications of thrombocytosis in acute phase Kawasaki disease. Eur. J. Pediatr..

[25] Ueno K., Nomura Y., Morita Y., Eguchi T., Masuda K., Kawano Y. (2015). Circulating platelet-neutrophil aggregates play a significant role in Kawasaki disease. Circ. J..

[26] Xing H., Tian G. (2020). Increased Interleukin-35 suppresses peripheral CD14+ monocytes function in patients with Kawasaki disease. BMC Immunol..

[27] Brogan P.A., Shah V., Clarke L.A., Dillon M.J., Klein N. (2008). T cell activation profiles in Kawasaki syndrome. Clin. Exp. Immunol..

[28] Brown T.J., Crawford S.E., Cornwall M.L., Garcia F., Shulman S.T., Rowley A.H. (2001). CD8 T lymphocytes and macrophages infiltrate coronary artery aneurysms in acute Kawasaki disease. J. Infect. Dis..

[29] Ehara H., Kiyohara K., Izumisawa Y., Ito T. (2010). Early activation does not translate into effector differentiation of peripheral CD8 T cells during the acute phase of Kawasaki disease. Cell. Immunol..

[30] Wang Y., Wang W., Gong F., Fu S., Zhang Q., Hu J., Qi Y., Xie C., Zhang Y. (2013). Evaluation of intravenous immunoglobulin resistance and coronary artery lesions in relation to Th1/Th2 cytokine profiles in patients with Kawasaki disease. Arthritis Rheum..

[31] Jia S., Li C., Wang G., Yang J., Zu Y. (2010). The T helper type 17/regulatory T cell imbalance in patients with acute Kawasaki disease. Clin. Exp. Immunol..

[32] Rasouli M., Heidari B., Kalani M. (2014). Downregulation of Th17 cells and the related cytokines with treatment in Kawasaki disease. Immunol. Lett..

[33] Ni F.F., Li C.R., Li Q., Xia Y., Wang G.B., Yang J. (2014). Regulatory T cell microRNA expression changes in children with acute Kawasaki disease. Clin. Exp. Immunol..

[34] Guo M.M.H., Tseng W.N., Ko C.H., Pan H.M., Hsieh K.S., Kuo H.C. (2015). Th17- and Treg-related cytokine and mRNA expression are associated with acute and resolving Kawasaki disease. Allergy.

[35] Ding Y., Li G., Xiong L.J., Yin W., Liu J., Liu F., Wang R.G., Xia K., Zhang S.L., Zhao L. (2015). Profiles of responses of immunological factors to different subtypes of Kawasaki disease. BMC Musculoskelet. Disord..

[36] Sugahara-Tobinai A., Inui M., Metoki T., Watanabe Y., Onuma R., Takai T., Kumaki S. (2019). Augmented ILT3/LILRB4 expression of peripheral blood antibody-secreting cells in the acute phase of Kawasaki disease. Pediatr. Infect. Dis. J..

[37] Xu M., Jiang Y., Wang J., Liu J., Liu C., Liu D., Yang S. (2019). Distinct variations of antibody-secreting cells and memory B cells during the course of Kawasaki disease. BMC Immunol..

[38] Xu M., Jiang Y., Zhang J., Zheng Y., Liu D., Guo L., Yang S. (2018). Variation in IL-21-secreting circulating follicular helper T cells in Kawasaki disease. BMC Immunol..

[39] Xu M., Jiang Y., Wang J., Liu D., Wang S., Yi H., Yang S. (2019). Distribution of distinct subsets of circulating T follicular helper cells in Kawasaki disease. BMC Pediatr..

[40] Rivas M.N., Arditi M. (2020). Kawasaki disease: Pathophysiology and insights from mouse models. Nat. Rev. Rheumatol..

[41] Zhu Y.P., Shamie I., Lee J.C., Nowell C.J., Peng W., Angulo S., Le L.N., Liu Y., Miao H., Xiong H. (2021). Immune response to intravenous immunoglobulin in patients with Kawasaki disease and MIS-C. J. Clin. Investig..

[42] Si F., Wu Y., Gao F., Feng S., Liu R., Yi Q. (2017). Relationship between IL-27 and coronary arterial lesions in children with Kawasaki disease. Clin. Exp. Med..

[43] Teraura H., Kotani K., Minami T., Takeshima T., Shimooki O., Kajii E. (2017). The serum concentration of soluble interleukin-2 receptor in patients with Kawasaki disease. Ann. Clin. Biochem..

[44] Ye Q., Shao W., Shang S., Zhang T., Hu J., Zhang C.C. (2015). A comprehensive assessment of the value of laboratory indices in diagnosing Kawasaki disease. Arthritis Rheumatol..

[45] Wu Y., Liu F.F., Xu Y., Wang J.J., Samadli S., Wu Y.F., Liu H.H., Chen W.X., Luo H.H., Zhang D.D. (2019). Interleukin-6 is prone to be a candidate biomarker for predicting incomplete and IVIG non-responsive Kawasaki disease rather than coronary artery aneurysm. Clin. Exp. Med..

[46] Li Y., Zheng Q., Zou L., Wu J., Guo L., Teng L., Zheng R., Jung L.K.L., Lu M. (2019). Kawasaki disease shock syndrome: Clinical characteristics and possible use of IL-6, IL-10 and IFN-γ as biomarkers for early recognition. Pediatr. Rheumatol..

[47] Nandi A., Pal P., Basu S. (2019). A comparison of serum IL6 and CRP levels with respect to coronary changes and treatment response in Kawasaki disease patients: A prospective study. Rheumatol. Int..

[48] Wang Y., Qian S.Y., Yuan Y., Wang Q., Gao L., Chen X., Yu X., Zhen Z. (2020). Do cytokines correlate with refractory Kawasaki disease in children?. Clin. Chim. Acta.

[49] Kim H.J., Choi E.H., Kil H.R. (2014). Association between adipokines and coronary artery lesions in children with Kawasaki Disease. J. Korean Med. Sci..

[50] Dai L., Zhang L., He J., Huang R., Tang W., Guo H., Shang X. (2024). Diagnostic value of syndecan-1 for coronary artery lesions and correlation analysis of laboratory indicators in Kawasaki disease patients. Ital. J. Pediatr..

[51] Fujimaru T., Ito S., Masuda H., Oana S., Kamei K., Ishiguro A., Kato H., Abe J. (2014). Decreased levels of inflammatory cytokines in immunoglobulin-resistant Kawasaki disease after plasma exchange. Cytokine.

[52] Hachiya A., Kobayashi N., Matsuzaki S., Takeuchi Y., Akazawa Y., Shigemura T., Motoki N., Masumoto J., Agematsu K. (2018). Analysis of biomarker serum levels in IVIG and infliximab-refractory Kawasaki disease patients. Clin. Rheumatol..

[53] Netea S.A., Biesbroek G., van Stijn D., Ijspeert H., van der Made C.I., Jansen M.H., Geissler J., van den Berg J.M.M., van der Kuip M., Gruppen M.P. (2023). Transient anti-cytokine autoantibodies superimpose the hyperinflammatory response in Kawasaki disease and multisystem inflammatory syndrome in children: A comparative cohort study on correlates of disease. EBioMedicine.

[54] Lin I.C., Suen J.L., Huang S.K., Chou M.H., Kuo H.C., Lo M.H., Kuo K.C., Wang L. (2024). Involvement of IL-17A/IL-17 receptor A with neutrophil recruitment and the severity of coronary arteritis in Kawasaki disease. J. Clin. Immunol..

[55] Tseng W.N., Lo M.H., Guo M.M.H., Hsieh K.S., Chang W.C., Kuo H.C. (2014). IL-31 associated with coronary artery lesion formation in Kawasaki disease. PLoS ONE.

[56] Brodeur K.E., Liu M., Ibanez D., de Groot M.J., Chen L., Du Y., Seyal E., Laza-Briviesca R., Baker A., Chang J.C. (2024). Elevation of IL-17 cytokines distinguishes Kawasaki disease from other pediatric inflammatory disorders. Arthritis Rheumatol..

[57] Zhao J., Xu Y., Shi C., Chai H., Shen Y., Ma X., Liu Y. (2025). Expression of serum ferritin, human neutrophil lipocalin, procalcitonin, and inflammatory factors in children with Kawasaki disease and their relationship to coronary artery lesions. Am. J. Transl. Res..

[58] Jinkawa A., Shimizu M., Nishida K., Kaneko S., Usami M., Sakumura N., Irabu H., Takakuwa M., Inoue N., Mizuta M. (2019). Cytokine profile of macrophage activation syndrome associated with Kawasaki disease. Cytokine.

[59] Weng K.P., Hsieh K.S., Huang S.H., Ou S.F., Lai T.J., Tang C.W., Lin C.C., Ho T.Y., Liou H.H., Ger L.P. (2013). Interleukin-18 and coronary artery lesions in patients with Kawasaki disease. J. Chin. Med. Assoc..

[60] Qi Y., Xu J., Lin Z., Tao Y., Zheng F., Wang Y., Sun Y., Fu S., Wang W., Xie C. (2021). The network of pro-inflammatory factors CD147, DcR3, and IL-33 in the development of Kawasaki disease. J. Inflamm. Res..

[61] Ko T.M., Kuo H.C., Chang J.S., Chen S.P., Liu Y.M., Chen H.W., Tsai F.J., Lee Y.C., Chen C.H., Wu J.Y. (2015). CXCL10/IP-10 is a biomarker and mediator for Kawasaki disease. Circ. Res..

[62] Su Y., Feng S., Luo L., Liu R., Yi Q. (2019). Association between IL-35 and coronary arterial lesions in children with Kawasaki disease. Clin. Exp. Med..

[63] Jia C., Zhuge Y., Zhang S., Ni C., Wang L., Wu R., Niu C., Wen Z., Rong X., Qiu H. (2021). IL-37b alleviates endothelial cell apoptosis and inflammation in Kawasaki disease through IL-1R8 pathway. Cell Death Dis..

[64] Matsuguma C., Wakiguchi H., Suzuki Y., Okada S., Furuta T., Ohnishi Y., Azuma Y., Ohga S., Hasegawa S. (2019). Dynamics of immunocyte activation during intravenous immunoglobulin treatment in Kawasaki disease. Scand. J. Rheumatol..

[65] Hu P., Jiang G.M., Wu Y., Huang B.Y., Liu S.Y., Zhang D.D., Xu Y., Wu Y.F., Xia X., Wei W. (2017). TNF-α is superior to conventional inflammatory mediators in forecasting IVIG non-response and coronary arteritis in Chinese children with Kawasaki disease. Clin. Chim. Acta.

[66] Kato M., Ayusawa M., Watanabe H., Komori A., Abe Y., Nakamura T., Kamiyama H., Takahashi S. (2018). Cardiac function on 3-D speckle tracking imaging and cytokines in Kawasaki disease. Pediatr. Int..

[67] Shimizu M., Mizuta M., Usami M., Inoue N., Sakakibara Y., Yamada K., Konishi M., Ohta K., Yachie A. (2018). Clinical significance of serum soluble TNF receptor II level and soluble TNF receptor II/I ratio as indicators of coronary artery lesion development in Kawasaki disease. Cytokine.

[68] Heidari B., Amin R., Kashef S., Alyasin S., Moghtaderi M., Aminshahidi M., Kalani M. (2014). Expression of CD11b as an adhesion molecule on neutrophils in children with Kawasaki disease. Iran. J. Allergy Asthma Immunol..

[69] Kobayashi T., Kimura H., Okada Y., Inoue Y., Kobayashi T., Shinohara M., Morikawa A. (2007). Increased CD11b expression on polymorphonuclear leucocytes and cytokine profiles in patients with Kawasaki disease. Clin. Exp. Immunol..

[70] Zou Q.M., Li X.H., Song R.X., Xu N.P., Zhang T., Zhang M.M., Lin Y., Shi L., Fu J., Cui X.D. (2015). Early decreased plasma levels of factor B and C5a are important biomarkers in children with Kawasaki disease. Pediatr. Res..

[71] Song R.X., Zou Q.M., Li X.H., Xu N.P., Zhang T., Fu J., Cui X.D. (2016). Plasma MASP-1 concentration and its relationship to recovery from coronary artery lesion in children with Kawasaki disease. Pediatr. Res..

[72] Feng S., Yadav S.K., Gao F., Yi Q. (2015). Plasma levels of monokine induced by interferon-gamma/chemokine (C-X-X motif) ligand 9, thymus and activation-regulated chemokine/chemokine (C-C motif) ligand 17 in children with Kawasaki disease. BMC Pediatr..

[73] Hosaka S., Imagawa K., Yano Y., Lin L., Shiono J., Takahashi-Igari M., Hara H., Hayashi D., Imai H., Morita A. (2024). The CXCL10-CXCR3 axis plays an important role in Kawasaki disease. Clin. Exp. Immunol..

[74] Lee C.P., Huang Y.H., Hsu Y.W., Yang K.D., Chien H.C., Yu H.R., Yang Y.L., Wang C.L., Chang W.C., Kuo H.C. (2013). TARC/CCL17 gene polymorphisms and expression associated with susceptibility and coronary artery aneurysm formation in Kawasaki disease. Pediatr. Res..

[75] Huang J., Zhao C., Zhang S. (2024). Semaphorin 7A promotes endothelial permeability and inflammation via plexin C1 and integrin β1 in Kawasaki disease. BMC Pediatr..

[76] Huang J., Wu S., Cao S., Zhu X., Zhang S. (2020). Neutrophil-derived semaphorin 4D induces inflammatory cytokine production of endothelial cells via different plexin receptors in Kawasaki disease. BioMed Res. Int..

[77] Guo M., Fan S., Chen Q., Jia C., Qiu M., Bu Y., Tang W.H., Zhang Y. (2022). Platelet-derived microRNA-223 attenuates TNF-α induced monocytes adhesion to arterial endothelium by targeting ICAM-1 in Kawasaki disease. Front. Immunol..

[78] Reindel R., Bischof J., Kim K.Y., Orenstein J.M., Soares M.B., Baker S.C., Shulman S.T., Perlman E.J., Lingen M.W., Pink A.J. (2014). CD84 is markedly up-regulated in Kawasaki disease arteriopathy. Clin. Exp. Immunol..

[79] Shuai S., Zhang H., Zhang R., Tang M., Luo E., Yang Y., Gao Y., Yue S., Liang H., Cai J. (2023). Prediction of coronary artery lesions based on C-reactive protein levels in children with Kawasaki disease: A retrospective cohort study. J. Pediatr. (Rio J.).

[80] Xia Y., Tian X., Li Q., Nakajima T., Saito H., Terai M. (2017). Expression of FcγRs on monocytes among Kawasaki disease patients with coronary artery lesions. Int. Immunopharmacol..

[81] Kim K.Y., Ahn Y., Kim D.Y., Kim H.S., Kim D.S. (2016). Elevated serum YKL-40 levels in patients with Kawasaki disease. Biomarkers.

[82] Wakiguchi H., Hasegawa S., Suzuki Y., Kudo K., Ichiyama T. (2015). Relationship between T-cell HLA-DR expression and intravenous immunoglobulin treatment response in Kawasaki disease. Pediatr. Res..

[83] Chen Z., Zeng A., Yang P., Zhang J., Liu D., Li M., Jing F., Yi Q. (2025). Role of leukocyte-associated Ig-like receptor-1 in the pathogenesis of Kawasaki disease and coronary artery aneurysms. Immunol. Lett..

[84] Armaroli G., Verweyen E., Pretzer C., Kessel K., Hirono K., Ichida F., Okabe M., Cabral D.A., Foell D., Brown K.L. (2019). Monocyte-derived interleukin-1β as the driver of S100A12-induced sterile inflammatory activation of human coronary artery endothelial cells: Implications for the pathogenesis of Kawasaki disease. Arthritis Rheumatol..

[85] Yi L., Zhang J., Zhong J., Zheng Y. (2020). Elevated levels of platelet activating factor and its acetylhydrolase indicate high risk of Kawasaki disease. J. Interferon. Cytokine. Res..

[86] Wu Q., Weng R., Xu Y., Wang L., Huang Y., Yang J. (2021). Activin A suppresses peripheral CD8+ T lymphocyte activity in acute-phase Kawasaki disease. BMC Immunol..

[87] Si F., Lu Y., Wen Y., Chen T., Zhang Y., Yang Y. (2023). Cathelicidin (LL-37) causes expression of inflammatory factors in coronary artery endothelial cells of Kawasaki disease by activating TLR4-NF-κB-NLRP3 signaling. Immun. Inflamm. Dis..

[88] Bordea M.A., Costache C., Grama A., Florian A.I., Lupan I., Samasca G., Deleanu D., Makovicky P., Makovicky P., Rimarova K. (2022). Cytokine cascade in Kawasaki disease versus Kawasaki-like syndrome. Physiol. Res..

[89] Porritt R.A., Chase Huizar C., Dick E.J., Kumar S., Escalona R., Gomez A.C., Marek-Iannucci S., Noval Rivas M., Patterson J., Forsthuber T.G. (2021). Inhibition of IL-6 in the LCWE mouse model of Kawasaki disease inhibits acute phase reactant serum amyloid A but fails to attenuate vasculitis. Front. Immunol..

[90] Yeung R.S. (2010). Kawasaki disease: Update on pathogenesis. Curr. Opin. Rheumatol..

[91] Li J., Li D., Hu M., Huang H., Xu S., Li H. (2022). Red blood cell distribution width and tumor necrosis factor-α for the early prediction of coronary artery lesion in Kawasaki disease: A retrospective study. Eur. J. Pediatr..

[92] Stringer E., Yeung R.S.M. (2008). Pathogenesis of Kawasaki disease: The central role of TNF-α. Future Rheumatol..

[93] Alphonse M.P., Duong T.T., Tam S., Yeung R.S.M. (2023). Mercury increases IL-1β and IL-18 secretion and intensifies coronary arteritis in an animal model of Kawasaki disease. Front. Immunol..

[94] Kaneko S., Shimizu M., Shimbo A., Irabu H., Yokoyama K., Furuno K., Tanaka T., Ueno K., Fujita S., Iwata N. (2023). Clinical significance of serum cytokine profiles for differentiating between Kawasaki disease and its mimickers. Cytokine.

[95] Patel B., Silwal A., Eltokhy M.A., Gaikwad S., Curcic M., Patel J., Prasad S. (2024). Deciphering CD59: Unveiling its role in immune microenvironment and prognostic significance. Cancers.

[96] Sproston N.R., Ashworth J.J. (2018). Role of C-reactive protein at sites of inflammation and infection. Front. Immunol..

[97] Zandstra J., van de Geer A., Tanck M.W.T., van Stijn-Bringas Dimitriades D., Aarts C.E.M., Dietz S.M., van Bruggen R., Schweintzger N.A., Zenz W., Emonts M. (2020). Biomarkers for the discrimination of acute Kawasaki disease from infections in childhood. Front. Pediatr..

[98] Yang Y., Hu X. (2022). The predictive values of MMP-9, PLTs, ESR, and CRP levels in Kawasaki disease with cardiovascular injury. Evid.-Based Complement. Altern. Med..

[99] Hokibara S., Kobayashi N., Kobayashi K., Shigemura T., Nagumo H., Takizawa M., Yamazaki T., Agematsu K. (2016). Markedly elevated CD64 expression on neutrophils and monocytes as a biomarker for diagnosis and therapy assessment in Kawasaki disease. Inflamm. Res..

[100] Wu Q., Yang Z., Huang Y., Wang L., Weng R., Yang J. (2021). Effect of Activin A on activation status of monocytes in acute-phase Kawasaki disease. Clin. Exp. Med..

[101] Ashraf M.A., Nookala V. (2025). Biochemistry of platelet activating factor. StatPearls.

[102] Rife E., Gedalia A. (2020). Kawasaki disease: An update. Curr. Rheumatol. Rep..

[103] Shulman S.T., Rowley A.H. (2015). Kawasaki disease: Insights into pathogenesis and approaches to treatment. Nat. Rev. Rheumatol..

[104] Fang X. (2025). The clinical value of dynamic monitoring of complete blood count in predicting immunoglobulin resistance in Chinese children with Kawasaki disease. Sci. Rep..

[105] Furukawa S., Matsubara T., Jujoh K., Sasai K., Nakachi S., Sugawara T., Yabuta K., Kato H. (1990). Reduction of peripheral blood macrophages/monocytes in Kawasaki disease by intravenous gammaglobulin. Eur. J. Pediatr..

[106] Kim Y.S., Yang H.J., Kee S.J., Choi I., Ha K., Ki K.K., Jeong I.S., Cho H.J. (2021). The “intermediate” CD14+CD16+ monocyte subpopulation plays a role in IVIG responsiveness of children with Kawasaki disease. Pediatr. Rheumatol. Online J..

[107] Ma Y., Zhang J., Fan R. (2022). Efficacy of glucocorticoid plus intravenous immunoglobulin in children with immunoglobulin-insensitive Kawasaki disease. J. Healthc. Eng..

[108] McAlpine S.M., Roberts S.E., Heath J.J., Käsermann F., Issekutz A.C., Issekutz T.B., Derfalvi B. (2021). High dose intravenous IgG therapy modulates multiple NK cell and T cell functions in patients with immune dysregulation. Front. Immunol..

[109] Kong W.X., Ma F.Y., Fu S.L., Wang W., Xie C.H., Zhang Y.Y., Gong F.Q. (2019). Biomarkers of intravenous immunoglobulin resistance and coronary artery lesions in Kawasaki disease. World J. Pediatr..

[110] Zhang H., Song H.-B., Wang D.X., Deng H.Y., Sun W.L. (2022). Correlation between the level of inflammatory cytokines and prognosis in children with IVIG-sensitive Kawasaki disease and IVIG-resistant Kawasaki disease. Pak. J. Med. Sci..

[111] Zhang C., Zhang X., Shen J., Lu X., Zhang J., Chen S. (2020). Changes in peripheral blood neutrophils, lymphocytes and IL-10 in children with Kawasaki disease from different age groups undergoing intravenous immunoglobulin: A retrospective study. Mediat. Inflamm..

[112] Li G., Wang T., Gou Y., Zeng R., Liu D., Duan Y., Liu B. (2020). Value of C-reactive protein/albumin ratio in predicting intravenous immunoglobulin-resistant Kawasaki disease—A data from multi-institutional study in China. Int. Immunopharmacol..

[113] Portman M.A., Dahdah N.S., Slee A., Olson A.K., Choueiter N.F., Soriano B.D., Buddhe S., Altman C.A., EATAK Investigators (2019). Etanercept with IVIg for acute Kawasaki disease: A randomized controlled trial. Pediatrics.

[114] Ling J., Xie F., Zhou Q., Ouyang Q., Li L., Zhao W., Liu X. (2024). Case series on the efficacy and safety of tocilizumab in IVIG-resistant Kawasaki disease: A retrospective analysis of five patients. J. Inflamm. Res..

[115] Kessel C., Koné-Paut I., Tellier S., Belot A., Masjosthusmann K., Wittkowski H., Fuehner S., Rossi-Semerano L., Dusser P., Marie I. (2022). An immunological axis involving interleukin 1β and leucine-rich-α2-glycoprotein reflects therapeutic response of children with Kawasaki disease: Implications from the KAWAKINRA trial. J. Clin. Immunol..

[116] Nomura O., Fukuda S., Ota E., Ono H., Ishiguro A., Kobayashi T. (2021). Monoclonal antibody and anti-cytokine biologics for Kawasaki disease: A systematic review and meta-analysis. Semin. Arthritis Rheum..

[117] McCrindle B.W., Rowley A.H., Newburger J.W., Burns J.C., Bolger A.F., Gewitz M., Baker A.L., Jackson M.A., Takahashi M., Shah P.B. (2017). American Heart Association Rheumatic Fever, Endocarditis, and Kawasaki Disease Committee of the Council on Cardiovascular Disease in the Young; Council on Cardiovascular and Stroke Nursing; Council on Cardiovascular Surgery and Anesthesia; Council on Epidemiology and Prevention. Diagnosis, treatment, and long-term management of Kawasaki disease: A scientific statement for health professionals from the American Heart Association. Circulation.

[118] Koizumi K., Hoshiai M., Katsumata N., Toda T., Kise H., Hasebe Y., Kono Y., Sunaga Y., Yoshizawa M., Watanabe A. (2018). Infliximab regulates monocytes and regulatory T cells in Kawasaki disease. Pediatr. Int..

[119] Koizumi K., Hoshiai M., Moriguchi T., Katsumata N., Toda T., Kise H., Hasebe Y., Kono Y., Sunaga Y., Yoshizawa M. (2019). Plasma exchange downregulates activated monocytes and restores regulatory T cells in Kawasaki disease. Ther. Apher. Dial..

[120] Duan M., Geng Z., Gao L., Zhao Y., Li Z., Chen L., Kuosmanen P., Qi G., Gong F., Yu G. (2025). An interpretable machine learning-assisted diagnostic model for Kawasaki disease in children. Sci. Rep..

[121] Kobayashi T., Inoue Y., Takeuchi K., Okada Y., Tamura K., Tomomasa T., Kobayashi T., Morikawa A. (2006). Prediction of intravenous immunoglobulin unresponsiveness in patients with Kawasaki disease. Circulation.

[122] Egami K., Muta H., Ishii M., Suda K., Sugahara Y., Iemura M., Matsuishi T. (2006). Prediction of resistance to intravenous immunoglobulin treatment in patients with Kawasaki disease. J. Pediatr..

[123] Sano T., Kurotobi S., Matsuzaki K., Yamamoto T., Maki I., Miki K., Kogaki S., Hara J. (2007). Prediction of non-responsiveness to standard high-dose gamma-globulin therapy in patients with acute Kawasaki disease before starting initial treatment. Eur. J. Pediatr..

[124] Kuniyoshi Y., Tsujimoto Y., Banno M., Taito S., Ariie T., Takahashi N., Tokutake H., Takada T. (2023). Prediction models for intravenous immunoglobulin resistance in Kawasaki disease: A meta-analysis. Pediatrics.

[125] Piram M., Darce Bello M., Tellier S., Di Filippo S., Boralevi F., Madhi F., Meinzer U., Cimaz R., Piedvache C., Koné-Paut I. (2020). Defining the risk of first intravenous immunoglobulin unresponsiveness in non-Asian patients with Kawasaki disease. Sci. Rep..

[126] Sleeper L.A., Minich L.L., McCrindle B.M., Li J.S., Mason W., Colan S.D., Atz A.M., Printz B.F., Baker A., Vetter V.L. (2011). Pediatric Heart Network Investigators. Evaluation of Kawasaki disease risk-scoring systems for intravenous immunoglobulin resistance. J. Pediatr..

[127] Takeshita S., Kanai T., Kawamura Y., Yoshida Y., Nonoyama S. (2017). A comparison of the predictive validity of the combination of the neutrophil-to-lymphocyte ratio and platelet-to-lymphocyte ratio and other risk scoring systems for intravenous immunoglobulin (ivig)-resistance in Kawasaki disease. PLoS ONE.

[128] Mirata D., Tiezzi A.C., Buffoni L., Pagnini I., Maccora I., Marrani E., Mastrolia M.V., Simonini G., Giani T. (2025). Learning-based models for predicting IVIG resistance and coronary artery lesions in Kawasaki disease: A review of technical aspects and study features. Pediatr. Drugs.

